# Annotations

**Published:** 1896-03-21

**Authors:** 


### Annotations.

### The Notification of Measles.

In a recent report on an outbreak of measles, Dr. Sykes, Medical Officer of Health for St. Pancras, gives an excellent summary of the arguments for and against the notification of measles, and its reception into hospitals for infectious diseases. The inevitable conclusion, he says, is that in large towns the value of notification of measles is small unless accompanied by provision for hospital accommodation and disinfection, and this is a question of expense. He thinks that at least 3,000 beds would be required for London, even on the assumption that only 50 per cent. of the cases were dealt with. We need, however, hardly point out that in such calculations measles is looked at from a purely isolation point of view, and but small consideration is paid to the saving of life which might be effected by providing hospital treatment for that very small proportion of the cases in which the disease assumes a serious aspect Even a comparatively small number of beds, say 500 devoted to the treatment of measles might do an immensity of good in saving infants from the consequences of a disease which, while often harmless enough among the well-to-do, is extremely fatal among the children of the very poor.

### "Medical Temperance."

The phrase, "Medical temperance," has had a prominent place in newspapers and men's minds during the last week or two. Science, supposing her to be capable of feeling as well as of reasoning, cannot but experience some regret in finding so great a diversity of opinion among medical men on the alcohol question, and, by consequence, so "uncertain a sound of the trumpet" among those who ought to be the authoritative guides of the people in matters physiological. Ought alcohol to be used at all, except as a medicine, or ought it not? That is a plain question, and the public would be exceedingly glad of a plain and trustworthy answer to it. We are told that on this, as on other questions, "Doctors differ." Most certainly; but even their differences ought to admit of a rational explanation, and sound principles of practical conduct should be capable of extraction from them by sufficiently reasonable minds. Why do doctors differ so much on the alcohol question? Is the answer to be looked for in the alcohol, or in the doctors, or in neither exclusively, but in the general facts of human physiology? The truth seems to be that the answer must be sought, not in alcohol only, nor in doctors only, nor in the facts of physiology only, but in all three. Alcohol is admittedly a powerful stimulant; it is also safe and harmless if it be used only occasionally and in proper quantities. Concerning its use as a medicinal stimulant, there is practical unanimity in the medical profession, and throughout the community. The real "question of strife" is this: Is alcohol a food? The scientific and reasonable answer seems to be that to some people it is a food, but to other people it is not. The principal cause then of medical diversities of opinion is the diversity of physiological conditions among the people. That is where the difficulty lies. Every man of moderate intelligence soon finds out for himself whether or not alcohol is a food for him, and in what quantity it does him good if it be a food for him. Just as a man decides for himself whether or not he will have a religion, so does he decide for himself whether or not he will take alcohol. The physician's duty, then, is not to concern himself with preaching impossible doctrines, but to give early warning whenever alcohol is doing harm, and in doing this he will speak with all the greater force in proportion as he is known to be temperate in regard to this question.

### Public-houses and Sanitary Authorities.

As is well known, the sanitary authorities all over the country are entrusted with the duty of making bye-laws in respect of common lodging-houses, and no one is allowed to open or to keep a common lodging-house unless it has been inspected, approved, and registered. Certain associations have under the cloak of charity evaded the regulations made in this respect, and have, so far, successfully resisted the right claimed by the sanitary officials to inspect their premises except under a magistrate's order. As we have already pointed out, this is a great evil, and a very weak spot in our sanitary defences. Another weak spot has, however, recently been made manifest in the possibility of a publican turning his house into what may be practically a common lodging-house, in which he may do as he likes, in defiance of all sanitary regulations, so long as the licensing magistrates do not interfere. A case recently came before the magistrates at Edgware in which the Harrow Urban District Council opposed the renewal of the licence to a public-house, among other reasons, on the grounds that a portion of the premises had been used as a common lodging-house, and not as an inn, that this portion was not registered as a common lodging-house, and that it was not fit to be registered as such under the bye-laws of the District Council. Needless to say, the case fell through. No amount of proof that the house was being used as common lodging-houses are used took away from the fact that the premises were licensed by the magistrates, and were practically on all fours with the Hotel Métropole or any other licensed house in regard to the reception of guests. The landlord absolutely refused to be inspected by the sanitary officials, and he maintained his right; nevertheless, by the common sense of the magistrates he was compelled to put his house in order, for nothing can alter the fact that licensed houses are absolutely under the control of the magistrates who grant their licences. It is to be hoped that magistrates all over the country will recognise their responsibility in this matter, for it is sure to be the case that, as the bye-laws for the regulation of common lodging-houses increase in stringency, there will be an increasingly strong temptation thrown in the way of the lower class of publicans to devote a portion of their premises to the reception of people of exactly the same class as those who frequent common lodging-houses, but free from the regulations and independent of the inspection of the sanitary officials. Under such a system it is needless to say that great evils might result, and it is to be hoped that magistrates will take care that, by virtue of the police inspection to which these houses are all liable, their sanitary condition may be maintained at a point at least no lower than what is prescribed for common lodging-houses by the sanitary authorities of the various districts.

